# Structured, Small-group Hands-on Teaching Sessions Improve Pre-clerk Knowledge and Confidence in Point-of-care Ultrasound Use and Interpretation

**DOI:** 10.7759/cureus.3484

**Published:** 2018-10-23

**Authors:** Amir H Safavi, Qian Shi, Maylynn Ding, Maryam Kotait, Jason Profetto, Vian Mohialdin, Ari Shali

**Affiliations:** 1 Medical Education and Simulation, McMaster University, Hamilton, CAN; 2 Family Medicine, McMaster University, Hamilton, CAN; 3 Pathology and Molecular Medicine, McMaster University, Hamilton, CAN

**Keywords:** undergraduate physical exam skills, teaching tool, pre-post study, pocus, ume, preclerkship, clinical skills, knowledge acquisition, confidence, small group learning

## Abstract

Introduction

Many undergraduate medical education (UME) programs have begun adopting point-of-care ultrasound (PoCUS) curricula, reflecting the increasing ubiquity of this technique across medical specialties. The structures of international PoCUS curricula have been extensively studied. However, the efficacy of these curricula to increase knowledge and confidence in PoCUS is less well-studied. We investigated whether a structured, small-group PoCUS teaching session consisting of pre-defined learning objectives, an introductory presentation, and a mandatory hands-on scanning component would increase pre-clerk knowledge of and confidence in PoCUS theory, use, and interpretation.

Methods

A pre-post study was designed to assess changes in pre-clerk knowledge and confidence in PoCUS theory, use, and interpretation. Pre-clerks were recruited from the Hamilton campus of the Michael G. DeGroote School of Medicine at McMaster University. Pre-clerks were organized into four groups, with an average group size of seven learners. Two preceptors each taught two groups. Sessions included an introductory PowerPoint presentation and one-on-one preceptor-guided practice in identifying abdominal and genitourinary structures using PoCUS. Student responses on pre- and post-intervention surveys were analyzed to identify changes in knowledge and confidence. Student satisfaction with the teaching session was assessed from self-reported levels of agreement with satisfaction statements. The strengths and areas of improvement for the teaching sessions were identified from open-ended survey responses.

Results

Data from 27 students indicated a significant improvement in knowledge test scores (p < .05), with no significant differences between groups (F(3,23) = 0.64, p = n.s.) or between students with different preceptors (p = n.s.). Students’ confidence in PoCUS use and interpretation improved significantly (p < .05 for both), with no significant differences between groups (F(3,23) = 0.70, p = n.s. and F(3,23) = 0.32, p = n.s., respectively) or between students with different preceptors (p = n.s. for both). Improvements in knowledge of and confidence in PoCUS use were significantly correlated (r = .44, p < .05). All of the students agreed that they liked the instruction, content, and structure of the teaching session. The most frequently cited strengths of the teaching sessions were the mandatory individual practice time per student, individualized instruction from and interactions with preceptors, and the small group structure of the sessions.

Conclusion

This study provides novel evidence that a structured, small-group teaching session featuring a didactic presentation, defined learning objectives, and mandatory hands-on learning can effectively teach introductory PoCUS knowledge and skills to pre-clerks and increase student confidence. Future studies will investigate the retention and application of PoCUS knowledge and skill throughout clerkship and early residency training to determine if this teaching model can facilitate longitudinal PoCUS learning and competency as well as improved diagnostic capabilities as students advance through undergraduate medical training.

## Introduction

Point-of-care ultrasound (PoCUS) refers to focused, portable ultrasound imaging performed and interpreted by a physician at the patient’s bedside to rule in or out specific pathologies [1]. Over the past decade, the application of PoCUS has expanded rapidly, largely due to decreasing costs and advances in technology portability and image quality. Today, PoCUS is a well-established practice in many settings across multiple medical specialties [2]. Consequently, many undergraduate medical education (UME) programs have begun adopting PoCUS curricula. A survey published in 2016 indicated that six of 17 Canadian medical schools had integrated bedside ultrasound into their undergraduate curricula [3].

The structure and content of UME PoCUS curricula have been extensively reviewed [4]. Most programs worldwide use a combination of didactic lectures supplemented with demonstration and/or hands-on scanning sessions to teach PoCUS to undergraduate medical students [4]. Most are guided by predefined learning objectives [5-6]. In Canada, the College of Medicine of the University of Saskatchewan has implemented a comprehensive ultrasound curriculum that spans the pre-clerkship and clerkship years, features didactic teaching and hands-on learning integrated with both the anatomy and clinical skills curricula, and is informed by learning objectives [7]. Studies have shown students prefer a combination of teaching modalities to learn ultrasound but benefit most from face-to-face, small-group instruction [8-10].

Despite the growing literature on the structure and content of UME PoCUS curricula, there continues to be a dearth of evidence supporting the effectiveness of these methods in teaching fundamental PoCUS knowledge and skills to undergraduate medical students. Several studies have shown that UME PoCUS teaching improves student competence and confidence when applying PoCUS knowledge and skill in the clinical setting [11-16], but there is a lack of data regarding the effectiveness of UME PoCUS teaching in improving the acquisition of rudimentary knowledge and confidence amongst pre-clerkship medical students. The purpose of this study was to determine whether a structured, small-group PoCUS teaching session consisting of predefined learning objectives, an introductory presentation, and a mandatory hands-on scanning component would increase pre-clerk knowledge of and confidence in PoCUS theory, use, and interpretation.

## Materials and methods

Setting and participants

Pre-clerks were recruited from the Hamilton campus of the Michael G. DeGroote School of Medicine at McMaster University. One hundred and fifty students were enrolled as pre-clerks in the 2016-2017 academic year and were eligible for this study. All eligible pre-clerks had attended PoCUS teaching under the pre-existing anatomy curriculum at the medical school. Recruitment and participation in teaching sessions took place over a one-month period from May 28, 2017, to June 28, 2017. Pre-clerks were notified of the study and teaching session through the class Facebook discussion group. Participation in this study was voluntary and did not impact academic standing. Participants were divided into four groups of similar sizes. Teaching sessions were conducted at the Anatomy Lab in the Hamilton campus of the medical school.

Teaching session design

This teaching session was developed based on prior forms of PoCUS teaching within the medical school’s curriculum, which consisted of informal didactic teaching and optional student participation in hands-on scanning during anatomy class. The pre-existing curriculum did not have specific learning objectives or a predetermined amount of scanning time per student.

The length of each teaching session was 90 minutes. Sessions began with 15 minutes of didactic teaching using a PowerPoint presentation outlining session objectives, basic ultrasound physics, an introduction to probe orientation and imaging planes, as well as overviews of image acquisition and how to measure lengths. Each student was then required to spend eight minutes using the PoCUS machine to practice orienting the probe, identifying imaging planes, locating specific predetermined organs, and measuring the dimensions of each organ while receiving individualized feedback from the preceptor. Two students from each group volunteered to serve as standardized patients for the session, allowing every student to participate in scanning. The remaining time in each session was unstructured, allowing for students and preceptors to determine the best use of that time to meet the learning needs of the group.

Teaching sessions were designed to be taught to groups of seven pre-clerks, which is the average group size used in the medical school’s pre-clerkship clinical skills curriculum. The sessions were taught by two preceptors who teach the pre-existing PoCUS teaching sessions at the Hamilton campus. Each preceptor taught two groups of students.

Study design

The teaching session was evaluated using a Kirkpatrick Model framework [17], specifically Levels One and Two, as this was a preliminary study of the efficacy of this teaching session. A pre-post study was designed to assess the primary outcomes, which were changes in pre-clerk knowledge and confidence in PoCUS physics, technique, and image interpretation (Kirkpatrick Level Two) following participation in the teaching session. The secondary outcomes of this study were assessments of pre-clerk satisfaction with the teaching sessions, as well as an identification of the strengths of the sessions and areas for improvement (Kirkpatrick Level One).

Data collection

Participants were given paper copies of both the pre and post-intervention surveys (Appendices 1 and 2) at the beginning of the teaching session, with instructions to not examine the post-intervention survey until the completion of the teaching session. Survey responses were anonymous and each pair of surveys was pre-labeled with an alphanumeric identifier to preserve anonymity while identifying pairs of surveys completed by the same participant.

Participants answered six questions to establish baseline knowledge of PoCUS physics, technique, and image interpretation prior to the teaching session (Appendix 1). Following the teaching session, participants answered the same six knowledge questions (Appendix 2). Participants were asked to self-report their confidence level as percentages in using and interpreting PoCUS in both surveys. Participants selected their level of agreement with a series of statements about their satisfaction with the teaching session using a seven-point Likert scale (Appendix 2). Participants were asked open-ended questions to identify the strengths and areas of improvement for the teaching sessions.

Analysis

Anonymized data from the paper-based surveys was transcribed to Microsoft Excel (Microsoft Corp., Redmond, WA, US), which was used to perform a statistical analysis. Two-tailed Welch’s t-tests were used to assess the statistical significance of differences in the mean knowledge raw score of all students before and after the teaching session and the mean change in the knowledge score of students taught by different preceptors. A one-way analysis of variance (ANOVA) was performed to assess the statistical significance of the difference in the mean change in the knowledge score of students in different teaching groups. Two-tailed Welch’s t-tests were used to assess the statistical significance of differences in the mean confidence levels of all students before and after the teaching sessions in PoCUS use and interpretation and the mean changes in these confidence levels of students taught by different preceptors. One-way ANOVA was performed to assess the statistical significance of the differences in the mean change in the confidence levels of students in different teaching groups. Cohen’s d formula was used to calculate the effect sizes of statistically significant differences. A Pearson correlation coefficient was calculated to determine if there is a linear correlation between changes in knowledge and confidence in PoCUS use or interpretation. “Agree” and “Strongly agree” responses were grouped to determine the percentage of students who were in favor of the satisfaction statements. Qualitative data were analyzed using a frequency analysis to identify the most common themes in the responses.

Ethics board review

This protocol was reviewed by the research ethics officer of the Hamilton Integrated Research Ethics Board and was exempted from review in accordance with the 2014 Tri-Council Policy Statement (TCPS2), Article 2.5, as it involves program development and evaluation related to enhancing the learning of point-of-care ultrasound.

## Results

One hundred and fifty students were enrolled in pre-clerkship at the Hamilton campus for the 2016-2017 academic year. From this group, 27 students volunteered to participate in this study, filling 96% of the spots available (28) for this study. While the study was designed to have seven students in each of the four teaching sessions, each group had a different number of students (seven, nine, six, and five, respectively) due to changes in student availability. All 27 students completed pre- and post-intervention surveys (100% response rate).

**Figure 1:**
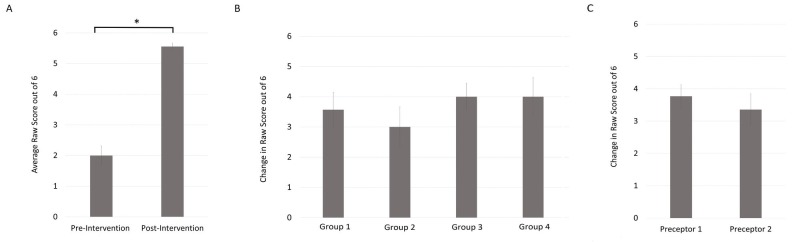
Teaching session improves student knowledge in PoCUS use and interpretation Data from 27 students indicated a significant improvement in knowledge test scores (*p < .05) (A), with no significant differences between groups (F(3,23) = 0.64, p = n.s.) (B) or between students with different preceptors (p=n.s.) (C). Error bars indicate the mean ± standard error. PoCUS: point-of-care ultrasound

Students obtained an average raw score of 2 out of 6 (SE 0.31, n = 27) on the pre-intervention knowledge test. Post-intervention, students obtained an average raw score of 5.56 (SE 0.1, n = 27) on the knowledge test. There was a 59.3% absolute increase in the knowledge score, which was a significant improvement (p < .05). Cohen’s effect size value (d = 0.89) was large and suggestive of high practical significance. There were no significant differences in the change in raw scores between the groups of students who participated in different teaching sessions (F(3,23) = 0.64, p = n.s.) or between students with different preceptors (p = n.s.) (Figure 1).

**Figure 2:**
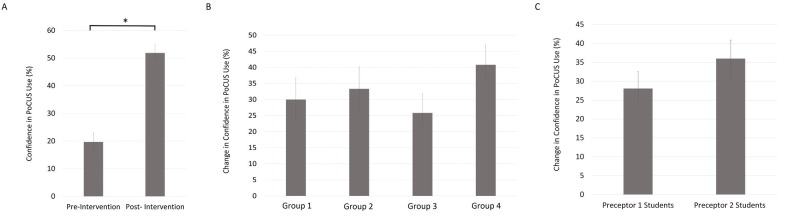
Teaching sessions improve student confidence in PoCUS use Data from 27 students indicated that students’ confidence in PoCUS use improved significantly (*p < .05) (A), with no significant differences between groups (F(3,23) = 0.70, p = n.s.) (B) or between students with different preceptors (p = n.s.) (C). Error bars indicate the mean ± standard error. PoCUS: point-of-care ultrasound

Prior to the intervention, the average level of confidence in PoCUS use amongst students was 19.67% (SE 3.51, n = 27). This level significantly increased (p < .05) to 51.85% (SE 3.09, n = 27) after the intervention, which is an absolute increase of 32.18%. Cohen’s effect size value (d = 1.91) was large and suggestive of high practical significance. There were no significant differences in this change in confidence between the groups of students in different teaching sessions (F(3,23) = 0.70, p = n.s.) or between students with different preceptors (p = n.s.) (Figure 2).

**Figure 3:**
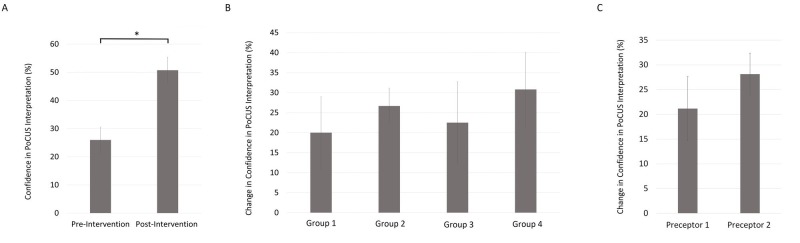
Teaching session improves student confidence in PoCUS interpretation Data from 27 students indicated that students’ confidence in PoCUS interpretation improved significantly (*p < .05) (A), with no significant differences between groups (F(3,23) = 0.32, p = n.s.) (B) or between students with different preceptors (p = n.s.) (C). Error bars indicate the mean ± standard error. PoCUS: point-of-care ultrasound

The pre-intervention average level of confidence in PoCUS interpretation amongst students was 25.96% (SE 4.57, n = 27). This confidence level significantly increased (p < .05) to 50.74% (SE 4.61, n = 27) post-intervention, which is an absolute increase of 24.78%. Cohen’s effect size value (d = 1.06) was large and suggestive of high practical significance. There were no significant differences in the change in confidence between the students in different teaching sessions (F(3,23 = 0.32, p = n.s.) or between students with different preceptors (p = n.s.) (Figure 3).

**Figure 4:**
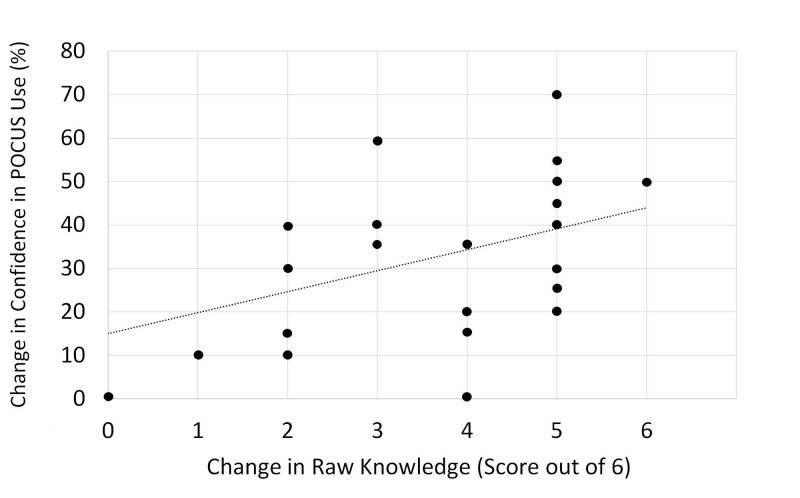
Post-teaching session increases in knowledge of and confidence in PoCUS use are correlated Pearson correlation analysis shows a significant moderate positive correlation between changes in confidence in PoCUS use and changes in knowledge test raw scores (r=.44; p<.05, n=27). Line represents linear regression of data (y=4.8282x + 15.018; r2=0.1905). PoCUS: point-of-care ultrasound

A Pearson correlation analysis indicated a moderate positive correlation between changes in confidence in PoCUS use and changes in knowledge test raw score, which was significant (r = .44; p < .05, n = 27) (Figure 4).

**Figure 5:**
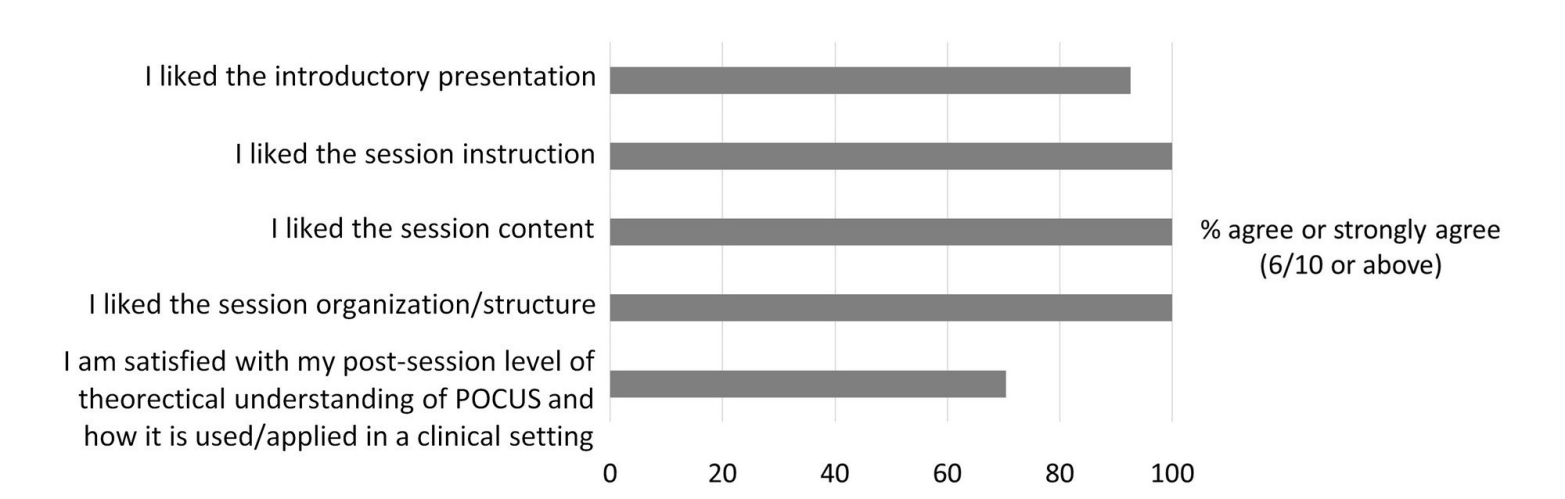
Student satisfaction survey results All 27 students agreed or strongly agreed that they liked the session instruction, content, and structure. 92.6% of students agreed or strongly agreed that they like the introductory presentation. 70.4% agreed or strongly agreed that they were satisfied with their post-session understanding of PoCUS theory and use. PoCUS: point-of-care ultrasound

Post-intervention, 92.6% of students agreed or strongly agreed that “(they) liked the introductory presentation, 100% agreed or strongly agreed that “(they) liked the session instruction,” 100% agreed that “(they) liked the session content,” 100% agreed or strongly agreed that “(they) liked the session organization/structure,” and 70.4% agreed or strongly agreed that “(they were) satisfied with their level of theoretical understanding of PoCUS and how it is used/applied in a clinical setting” (Figure 5).

**Figure 6:**
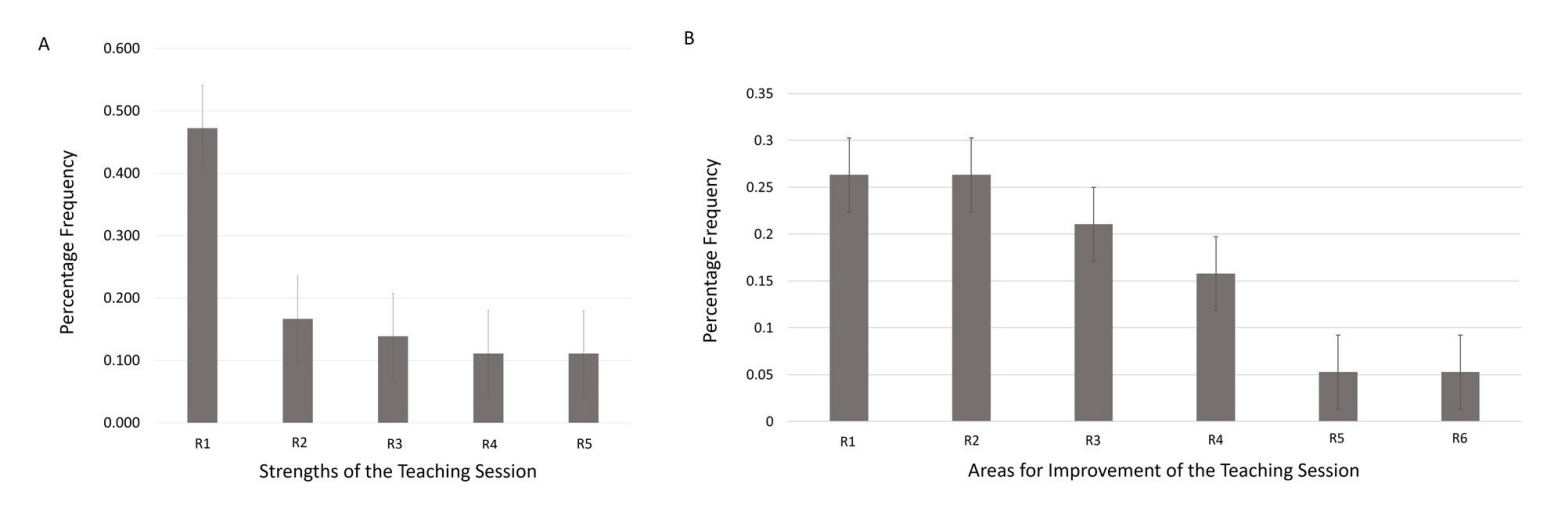
Student-reported strengths and areas for improvement of the teaching sessions Figure 6A indicates the following student-reported strengths of the teaching sessions, along with the relative frequency of these strengths amongst student responses: required individual practice time per student by 47.2% (A-R1), individualized instruction from and interactions with preceptors by 16.7% (A-R2), small group size (A-R3), relevance to pre-existing anatomy curriculum (A-R4), structure and organization of the session (A-R5). Figure 6B indicates the following areas for improvement of the teaching sessions, along with the relative frequency of these areas amongst student responses: increase the frequency of sessions per medical foundation (B-R1), provide pre- and post-session educational resources for further enrichment (B-R2), include more realistic images and/or videos in introductory slides (B-R3), smaller groups and more one-on-one student-to-preceptor practice time (B-R4), more clinically relevant content (B-R5), and more relevant anatomy content (B-R6).

A frequency analysis of the qualitative data gathered in the post-intervention survey indicates that the most frequently cited strength of the session was the required individual practice time per student. Other strengths of the sessions noted by students included the individualized instruction from and interactions with preceptors, as well as the small group structure of the sessions. The two most frequently suggested changes were to provide pre- and post-session educational resources to enrich the session experiences and to increase the frequency of sessions per medical foundation (more than one per medical foundation) (Figure 6).

## Discussion

While the structures of international UME PoCUS curricula have been extensively reviewed, the efficacy of these curricula to increase knowledge and confidence in PoCUS theory, use, and interpretation is less well-studied. Furthermore, there are no studies that have examined the efficacy of UME PoCUS curricula in improving rudimentary knowledge acquisition and student confidence amongst pre-clerks at Canadian medical schools. In this study, we aimed to determine whether a structured, small group teaching session consisting of an introductory presentation, pre-defined learning objectives, and mandatory hands-on learning would increase pre-clerk knowledge of and confidence in PoCUS theory, use, and interpretation. Our findings demonstrate that this teaching session can lead to significant increases in the knowledge of and confidence in PoCUS use and interpretation and, therefore, is an effective introductory session on PoCUS to pre-clerks.

This teaching session led to significant absolute increases in PoCUS knowledge (59.3%), confidence in PoCUS use (32.18%), and confidence in PoCUS interpretation (24.78%), with a moderate positive correlation (r = .44) between the former two changes. Furthermore, the Cohen’s d effect sizes of these increases (0.89, 1.91, 1.06) were large, suggesting that they were not only statistically significant but also meaningful changes with high practical significance. Thus, this teaching session may be an effective way of teaching introductory PoCUS knowledge and skills to pre-clerks and developing their confidence in PoCUS use and interpretation. There were no statistically significant differences in the changes in knowledge and confidence between students taught by different preceptors or between student groups. This may suggest that the effectiveness of this study was not influenced by the variability in teaching style and ability, group size, or dynamic interactions occurring within groups and that the structure and design of this teaching session lead to improved knowledge and confidence in PoCUS use and interpretation in the population studied.

A review of the literature suggests that there are no other published studies evaluating how effective an introductory PoCUS teaching session is at increasing knowledge and confidence in PoCUS use and interpretation amongst pre-clerk medical students. This study provides novel evidence that a structured, small-group teaching session featuring a didactic presentation, defined learning objectives, and mandatory hands-on learning can effectively teach introductory PoCUS knowledge and skills to pre-clerks and increase student confidence. In addition, students expressed satisfaction with the teaching session and identified multiple strengths of this approach to teaching PoCUS to pre-clerks. As a result of this study, this teaching session is now used by McMaster University’s Michael G. DeGroote School of Medicine to teach introductory PoCUS sessions to pre-clerks.

There are several limitations to this study. Given the availability of PoCUS machines and pre-clerks, this study had the capacity to recruit only 28 participants, representing 19% of the pre-clerks at the Hamilton campus, and 27 students ultimately participated in the study. The results of this study may be impacted by a self-selection bias, as the students were recruited through a call for student participation in a session providing additional PoCUS teaching. The selection bias may also have been introduced by posting the call for student participation on the class Facebook discussion group; all eligible pre-clerks were members of this page and had equal access to this announcement, but those not using Facebook regularly may have missed the opportunity to participate in this study. Statistically significant differences with large effect sizes were noted in knowledge and confidence in PoCUS use and interpretation, but the effect sizes may have been inflated due to the small sample size and low power of the study. This small-sample study may have lacked adequate power to detect significant differences in outcomes between students taught by different instructors and between student groups.

While this study indicated a significant improvement in pre-clerk knowledge and confidence in PoCUS use and interpretation, it was not designed to determine if it is superior to any pre-existing teaching methods, as there was an insufficient number of PoCUS machines and pre-clerks in order to study control groups. Furthermore, this study did not assess the retention and application of PoCUS knowledge, procedural skill, or confidence in clinical settings (Kirkpatrick Level Three) [17]. Future studies using this teaching session should recruit all pre-clerks enrolled at a medical school, utilize a randomized control trial study design, and incorporate longitudinal assessments throughout clerkship rotations in order to study the retention and application of PoCUS knowledge, skill, and confidence.

In addition, this study only recruited students from one medical school. These results may not be generalizable to all Canadian medical students, as there could be a significant variation in the knowledge of pre-clerks at the same stage of training and a spectrum of learner perspectives, which may not have been captured in this study.

In lieu of a standardized set of questions designed to assess the knowledge of PoCUS theory, image orientation, and procedural skills, we developed six questions to measure pre-clerk knowledge before and after the intervention. These questions were developed based on content taught in the introductory session of the pre-existing PoCUS curriculum at the medical school and in collaboration with the preceptors who taught both the pre-existing curriculum as well as the teaching sessions of the study. While the content of these questions was internally validated, the questions were not externally validated by a committee of PoCUS experts.

This study recruited pre-clerks who had received prior PoCUS teaching under the pre-existing curriculum at the medical school. Our results suggest that this teaching session can significantly improve the knowledge of PoCUS and confidence in the use and interpretation amongst pre-clerks who have already received prior introductory PoCUS teaching. This study should be repeated with pre-clerks without prior PoCUS learning to determine if it is an effective introductory PoCUS teaching session for pre-clerks learning this material for the first time.

## Conclusions

PoCUS is becoming an increasingly ubiquitous, clinical, decision-making tool in a multitude of specialties. As demand for proficiency in PoCUS increases, trainees will be expected to in turn improve their proficiency with the PoCUS technique and image interpretation. While several studies have suggested that incorporating PoCUS in UME improves student confidence and competence in the clinical setting, few have evaluated the effectiveness of PoCUS teaching in improving the acquisition of rudimentary knowledge and confidence amongst pre-clerkship medical students. This structured, small-group PoCUS teaching session, consisting of pre-defined learning objectives, an introductory presentation, and a mandatory hands-on scanning component, increased pre-clerk knowledge of and confidence in PoCUS theory, use, and interpretation. Future studies will investigate the retention and application of knowledge and skill throughout clerkship and early residency training to determine if this teaching model can facilitate longitudinal PoCUS learning and competency as well as improved diagnostic capabilities as students advance through undergraduate medical training.

## Figures and Tables

**Figure 7:**
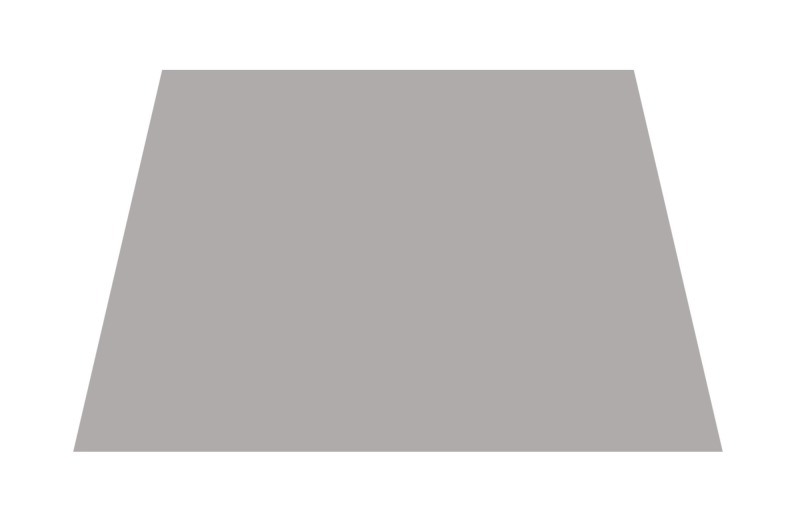
Image orientation question

## Appendices

Appendix 1: pre-session survey

Knowledge Questions

1. _____________ is the term referring to the brightness or darkness of a structure (relative to its

surroundings) on an ultrasound image.

2. If the ultrasound probe marker is pointing to the patient’s right, please indicate the orientation of the resultant image by labeling the sides of the following image (Figure 7):

3. You would like to obtain a transverse scan of a patient’s liver. The patient is supine on their back.

a) List the steps you would take to do this.

b) How would you obtain a sagittal view?

4. The difference in echogenicity between structures is due to the __________________ at the

interface (answer requires more than one word).

5. If the ultrasound probe marker is pointing to the patient’s head, please indicate the orientation of the resultant image by labeling the sides of the following image (Figure 7).

6. Once you have identified an organ of interest, you wish to measure its length. Please list the steps you would take to determine the length.

Confidence Questions

My confidence in my ability to use PoCUS is ___________ % (range: 1%-100%).

My confidence in my ability to interpret and visualize anatomical structures using PoCUS images is ___________ % (range: 1-100%).

Appendix 2: post-session survey

Knowledge Questions

1. _____________ is the term referring to the brightness or darkness of a structure (relative to its surroundings) on an ultrasound image.

2. If the ultrasound probe marker is pointing to the patient’s right, please indicate the orientation of the resultant image by labeling the sides of the following image (Figure 7).

3. You would like to obtain a transverse scan of a patient’s liver. The patient is supine on their back.

a) List the steps you would take to do this.

b) How would you obtain a sagittal view?

4. The difference in echogenicity between structures is due to the __________________ at the interface (answer requires more than one word).

5. If the ultrasound probe marker is pointing to the patient’s head, please indicate the orientation of the resultant image by labeling the sides of the following image (Figure 7).

6. Once you have identified an organ of interest, you wish to measure its length. Please list the steps you would take to determine the length.

Confidence Questions

After this session, my confidence in my ability to use PoCUS is ___________ % (range: 1%-100%)

After this session, my confidence in my ability to interpret and visualize anatomical structures using PoCUS images is ___________ % (range: 1%-100%).

Satisfaction Statements

I liked the introductory presentation.

1                2                3                4                5                6                7             8             9             10

strongly                                                        neither disagree                                                strongly                   

disagree                                                             nor agree                                                     agree                          

I liked the session instruction.

1                2                3                4                5                6                7             8             9             10

strongly                                                        neither disagree                                                strongly                   

disagree                                                             nor agree                                                     agree                          

I liked the session content.

1                2                3                4                5                6                7             8             9             10

strongly                                                        neither disagree                                                strongly                   

disagree                                                             nor agree                                                     agree                          

I liked the session organization/structure.

1                2                3                4                5                6                7             8             9             10

strongly                                                        neither disagree                                                strongly                   

disagree                                                            nor agree                                                    agree                          

I am satisfied with my post-session level of theoretical understanding of PoCUS and how it is used/applied in a clinical setting.

1                2                3                4                5                6                7             8             9             10

strongly                                                        neither disagree                                                strongly                   

disagree                                                             nor agree                                                     agree                          

Strengths of the teaching session:

Areas of improvement of the teaching session:
